# Factors associated with a low prevalence of exclusive breastfeeding during hospital stay in urban and semi-rural areas of southern Vietnam

**DOI:** 10.1186/s13006-018-0188-3

**Published:** 2018-10-19

**Authors:** Quynh-Nhi Thi Le, Khanh-Lam Phung, Van-Thuy Thi Nguyen, Katherine L. Anders, Minh-Nguyet Nguyen, Diem-Tuyet Thi Hoang, Thuy-Tien Thi Bui, Vinh-Chau Van Nguyen, Guy E. Thwaites, Cameron Simmons, Stephen Baker

**Affiliations:** 1 The Hospital for Tropical Diseases, Wellcome Trust Major Overseas Programme, Oxford University Clinical Research Unit, Ho Chi Minh City, Vietnam; 2 University of Medicine and Pharmacy in Ho Chi Minh City, Ho Chi Minh City, Vietnam; 3 School of Biological Sciences, Monash University, Clayton, VIC Australia; 4 Hung Vuong Hospital, Ho Chi Minh City, Vietnam; 5 The Hospital for Tropical Diseases, Ho Chi Minh City, Vietnam; 6 Department of Microbiology and Immunology, University of Melbourne, Parkville, Australia; 7 Centre for Tropical Medicine, Nuffield Department of Clinical Medicine, Oxford University, Oxford, UK; 8 The Department of Medicine, University of Cambridge, Cambridge, UK

**Keywords:** Breastfeeding, Rural, Urban, Vietnam, Risk factor, C-section

## Abstract

**Background:**

There is a paucity of data regarding risk factors associated with suboptimal breastfeeding practices in urbanized areas of low-middle income countries (LMICs).

**Methods:**

Through a large prospective birth cohort, which enrolled 6706 infants in Vietnam between 2009 and 2013, we investigated the practice of exclusive breastfeeding during hospital stay in urban and semi-rural populations and aimed to identify factors associated with suboptimal breastfeeding practices. Univariate and multivariable logistic regression were performed to determine factors associated with not exclusive breastfeeding during hospital stay.

**Results:**

Of 6076 mothers, 33% (2187) breastfed their infant exclusively before hospital discharge; 9% (364/4248) in urban and 74% (1823/2458) in semi-rural areas. Exclusive breastfeeding up to 4 months was recorded in 15% (959/6210) of participants; this declined to < 1% (56/6093) at 6 months. Delivery by Caesarean section (Odds Ratio [OR] 0.07; 95% Confidence Interval [CI] 0.04, 0.11 and OR 0.05; 95% CI 0.03, 0.08) and neonatal complications (OR 0.2; 95% CI 0.07, 0.47 and OR 0.25; 95% CI 0.14, 0.46) were common and highly significant risk factors associated with a lack of exclusive breastfeeding during hospital stay in urban and semi-rural settings, respectively.

**Conclusions:**

To our knowledge, this is the first large-scale investigation aimed at identifying factors associated with exclusive breastfeeding during hospital stay in Vietnam. Breastfeeding promotion strategies should prioritize common risk factors in hospital, such as Caesarean section and neonatal complications, and other location specific factors associated with socioeconomics.

**Electronic supplementary material:**

The online version of this article (10.1186/s13006-018-0188-3) contains supplementary material, which is available to authorized users.

## Background

Optimum breastfeeding is proven to be effective and cost-efficient in preventing child mortality and morbidity [1]. Vietnam has observed a substantial improvement in child nutrition in the last decade and despite breastfeeding being a common social practice in Vietnam (98% mothers report breastfeed their children at some point [2]), it has been estimated than only 24% of Vietnamese mothers practice exclusive breastfeeding when their children are < 6 months old [3]. Infant formula is widely promoted and used in Vietnamese hospitals, with > 50% children fed infant formula during the first 3 days after birth [4]. Very few hospitals (9%) have been accredited to the full implementation of the Baby-Friendly Hospital Initiative (BFHI), which supports exclusive breastfeeding, despite this initiative being launched in 1994 [1, 5, 6].

Previous studies have shown that good breastfeeding practices in the first few months of life are associated with early and correct infant feeding after birth in the first few days and before hospital discharge [7]. Recent studies from Vietnam have measured the initiation of breastfeeding within 1 hour after birth, which is a common indicator in breastfeeding behavioural studies; the rates were: 47.5% in an urban southern area in 2000 [8], 73.6% in a rural northern area in 2002 [9], and 90.6% in an urban central area in 2017 [10]. Notably, there were locations where children born into less affluent households were subjected to early breastfeeding more commonly than children born into more affluent households [3]. In contrast, an additional study from northern Vietnam found a higher prevalence of initiating breastfeeding within 1 hour after delivery in urban areas in comparison to rural areas [11]. This observation was explained by a higher education level of mothers from urban areas. There are little accurate data regarding breastfeeding practices in low-middle income countries (LMICs) that are undergoing economic development and urbanization, such as Vietnam [3]. It is important to further investigate differences in breastfeeding practices and associated factors between different settings in such locations.

In 2008 we initiated a birth cohort for investigating determinants of infectious diseases in urban and semi-rural infant populations in southern Vietnam [12]. Here, using data from this cohort, we aimed to assess the prevalence of various breastfeeding practices, including exclusive breastfeeding, and any breastfeeding activity during hospital stay. We further aimed to explore patterns of infant feeding during the first year of life and identify risk factors associated with inadequate breastfeeding practices during hospital stay in urban and semi-rural areas. Understanding these factors may assist in providing supporting information for promoting health strategies for expectant mothers and their infants.

## Methods

### Study location

Mothers residing in District 8 of Ho Chi Minh City, in Cao Lanh City and in Cao Lanh District (in Dong Thap Province) were invited to join the study. Ho Chi Minh City is the largest city in southern Vietnam with population of 7,820,000 in 2013 and density of 3731 person/ km^2^ [13]. District 8 is an urban area within Ho Chi Minh City with population of > 400,000 people and density of > 20,000 person/ km^2^ [14]. Dong Thap is a semi-rural province in the Mekong Delta, located 165 km southwest of Ho Chi Minh City. Cao Lanh City, the provincial capital, had a population of 163,030 people and a population density of 1523 person/km^2^ in 2013; 44.7% of the population live in a rural setting [15]. Cao Lanh District is a larger geographical area than Cao Lanh City with a population of 202,117 people and a population density of 412 person/km^2^; 93.6% of the population live in a rural setting [15].

### Study design and participants

This was a prospective birth cohort; the study design has been described previously [12]. Recruitment was conducted at Hung Vuong Hospital (HVH) in Ho Chi Minh City and Dong Thap Hospital (DTH) in Dong Thap Province, between 2009 and 2013 by trained study nurses who were midwives employed by these hospitals. Mothers aged > 15 years residing in either Ho Chi Minh City or Dong Thap Province for at least 12 months were invited to participate in the study at the time of hospital admission for delivery or during their antenatal visit in the 9th month of pregnancy. After providing informed consent, mothers were enrolled; infants from enrolled mothers who delivered at study hospitals were also enrolled before hospital discharge at birth.

### Data collection and management

Data were collected through face-to-face interviews conducted by trained study nurses using a standardized electronic questionnaire. All study nurses were senior hospital staff with recent training (prior to conducting recruitment and interviews) in good clinical practice, standard operating procedures, and communication skills. Upon enrolment, the study nurses collected information regarding socioeconomics, obstetrics history, the characteristics of parents and infants, delivery information and current feeding practice; more detailed information can be found in the published protocol [12].

The study nurses additionally conducted routine follow up visits of enrolled infants in these hospitals at 2, 4, 6, 9, and 12 months of age. Infants in Ho Chi Minh City had additional visits at 1 and 3 months of age when they received a routine check-up and scheduled immunizations. At each follow up visit, we interviewed mothers or caregivers with questions regarding the health status of the infants, changes in demographic information, current feeding practice, and infectious disease. At 4, 9 and 12 months of age, blood samples (1 ml) were collected for serological testing against various viral pathogens. Data collected at enrolment and at routine follow-up visits were stored in an encrypted web-based database. When the data collection was finalised, all data was checked and corrected for errors. Variables were derived and coded according to pre-defined definitions to produce datasets suitable for analysis.

### Breastfeeding data collection

Infants were enrolled at birth and breastfeeding practices were assessed before hospital discharge using multiple-choice questions regarding whether infants were exclusively breastfed, breastfed partially, or formula-milk fed after delivery. At each routine follow up visit, breastfeeding practices were again assessed by using multiple-choice questions on whether infants were currently breastfed (within the last month), exclusively breastfed, or fed by combinations of breastmilk, formula-milk, and solid food. These current feeding practices were the self-reported behaviour that mothers provided at the interview.

### Main variable definitions

Self-reported breastfeeding practices in the cohort were collected at each routine interview as (i) “exclusive breastfeeding” if receiving breast milk only, or (ii) “partial breastfeeding” if receiving breast milk in combination with other types of food, or (iii) “no breastfeeding” if not receiving breast milk. Based on reported breastfeeding practice at each interview time point, we defined variables of breastfeeding practices included (1) “exclusive breastfeeding during hospital stay”, (2) “any breastfeeding during hospital stay”, (3) “any breastfeeding”, (4) “exclusive breastfeeding for 4 months” and (5) “exclusive breastfeeding for 6 months”. Exclusive breastfeeding (1) during hospital stay was defined as baby was fed breast milk only during hospital stay, (2) “any breastfeeding during hospital stay” was defined as either exclusive or partial breastfeeding before hospital discharge after delivery, (3) “any breastfeeding” was defined as either exclusive or partial breastfeeding at any time-point from birth to the last follow-up visit. Children were considered as (4) “exclusive breastfeeding for four months” and (5) “exclusive breastfeeding for six months” if their mothers reported exclusive breastfeeding at all interviews from birth to the 4 months and 6 months follow-up visits, respectively.

The selected outcome for the main analysis was exclusive breastfeeding during hospital stay. The explanation variables were pre-defined as those that could affect breastfeeding practice at birth. These variables included socioeconomic status (household income), general characteristics of the mothers (first live child, age, education with high education meaning > 9 years at school, occupation, ethnic group, marital status, living arrangements, complications during pregnancy, and infection with either HIV or Hepatitis B), general characteristics of the fathers (education, occupation and ethnic group), general characteristics of the infants (age at birth, sex, delivery method including Caesarean section or vaginal birth, low birthweight (< 2500 g), premature birth with gestational age at birth < 37 weeks, and neonatal complications). These variables were selected based on subject-matter knowledge and data availability. The Social Economic Status (SES) score was derived using the Demographic and Health Surveys Program using principle component analysis which was categorized into quintile levels [16].

### Statistical analysis

Data generated in Ho Chi Minh City and Dong Thap were analysed separately due to differences in context and follow-up schedules. As missing data in variables of interest were low, the maximum number of missing data per variable was 11 and the number of cases with at least one missing value (%) was 19/6706 (< 1%), we conducted a complete-case analysis. Descriptive analyses are presented by frequencies and proportions for categorical variables and medians and interquartile ranges for continuous variables. Comparisons between groups of participants were performed using the Kruskal-Wallis test for continuous variables, and Chi-squared or Fisher’s exact test for categorical variables. Univariable and multivariable logistic regression were performed to determine factors associated with not initiating exclusive breastfeeding at birth. These analyses were stratified by type of data collection on diarrheal diseases (follow-up and passive data), and urban/semi-rural areas. Statistical significance was defined as a *p* <  0.05; all analyses were performed using R statistical software [17].

## Results

### Demographic features of the study population

From 2009 to 2013, 7274 mothers were invited to participate this cohort study; 6743 (93%) completed the baseline enrolment interview. Ultimately, 6706 infants from 6679 mothers were enrolled in the birth cohort. With the exception of one mother, who was interviewed 9 days after delivery, the breastfeeding practices of all mothers/infants were assessed within the first 7 days after birth and 6413/6706 (96%) were assessed within 2 days of delivery. The follow-up rate was high, with 5307/6706 (79%) infants attending all follow-up visits; 1202/6706 (18%) infants missed several follow-up visits and 197/6706 (3%) of infants did not attend any follow-up visits. Common causes of attrition included living too far from the study location and a reluctance of having blood drawn. In comparison to those attending all follow-up visits, infants who missed follow-up visits were more likely to live in the semi-rural area, be from a family with a low household income, not be the first child in the household, have parents with lower level education, have mothers without complications during pregnancy, and born by vaginal delivery (Additional file 1). The majority of infants (5778/6706, 86%) attended the 12-month follow-up visit.

Sixty three percent (4248/6706) of participants were resident in the urban area (Ho Chi Minh City) and 37% (2458/6706) were resident in the semi-rural area (Dong Thap) (Table 1). Mothers in the urban area were generally of a higher social economic status (92% (3880/4247) in the three highest quintiles) in comparison to mothers in the semi-rural area (98% (2399/2455) in the two lowest quintiles). The majority of mothers were bearing a child for the first time (58%; 3863/6702) and had a median age of 27 years. Almost all mothers were of Kinh ethnicity, with only 5% belonging to other minorities; most (4%; 246/6701) minorities in the urban area were Chinese. The level of parents’ education was higher in the urban area than in the semi-rural area; 40% of mothers and 46% of fathers had > 9 years of education in the urban area compared respectively to only 23% and 28% in the semi-rural area. In addition, Caesarean sections were more common in the urban area than the rural area (40% vs. 7%, respectively). A comparable urban vs. rural trend was observed for maternal complications (22% vs. 13%), maternal infections (7% vs. 1%), delivery before week 37th (4% vs. 2%), and infant complications at birth (5% vs. 2%).

**Table 1:** The characteristics of the study participants

Characteristic	Total (*n* = 6706)		Urban (*n* = 4248)		Semi-rural (*n* = 2458)	
	*n*	Frequency (%)	*n*	Frequency (%)	*n*	Frequency (%)
Household income	6702		4247		2455	
1st quintile (lowest)		1338 (20)		47 (1)		1291 (53)
2nd quintile		1418 (21)		310 (7)		1108 (45)
3rd quintile		1713 (26)		1657 (39)		56 (2)
4th quintile		921 (14)		921 (22)		0 (0)
5th quintile (highest)		1312 (20)		1312 (31)		0 (0)
Mother						
Primiparous	6702	3863 (58)	4247	2371 (56)	2455	1492 (61)
Age ^a^ (years)	6702	27 (23, 31)	4245	28 (24, 32)	2457	25 (21, 29)
Ethnic	6701		4247		2454	
Kinh		6397 (95)		3948 (93)		2449 (99)
Chinese		246 (4)		246 (6)		0 (0)
Other		58 (1)		53 (1)		5 (< 1)
High education	6702	2253 (34)	4247	1692 (40)	2455	561 (23)
Currently married	6702	6611 (99)	4247	4171 (98)	2455	2440 (99)
In-paid employment mother	6702	4425 (66)	4247	2737 (64)	2455	1688 (69)
Living with others	6699	6529 (97)	4247	4095 (96)	2452	2434 (99)
Complication during pregnancy	6706	1259 (19)	4248	950 (22)	2458	309 (13)
HIV and/or Hepatitis B infection	6706	313 (5)	4248	290 (7)	2458	23 (1)
Father						
Ethnic	6695		4242		2453	
Kinh		6232 (93)		3784 (89)		2448 (99)
Chinese		401 (6)		401 (9)		0 (0)
Other		62 (1)		57 (1)		5 (< 1)
High education	6696	2629 (39)	4242	1931 (46)	2454	698 (28)
In-paid employment father	6696	6659 (99)	4242	4210 (99)	2454	2449 (100)
Infant						
Premature at birth	6706	199 (3)	4248	149 (4)	2458	50 (2)
Male	6706	3503 (52)	4248	2248 (53)	2458	1255 (51)
Caesarean section	6706	1877 (28)	4248	1715 (40)	2458	162 (7)
Low birthweight	6706	309 (5)	4248	199 (5)	2458	110 (4)
Neonatal complication at birth	6706	275 (4)	4248	218 (5)	2458	57 (2)

^a^described in median (interquartile)

High education: completed lower secondary school (> 9 years of education), Premature at birth: gestational age at birth < 37 weeks, Low birthweight: birthweight < 2500 g

### Breastfeeding practices

The majority of mothers (91%; 6106/6706) fed their infants with breast milk on at least one occasion during the period in hospital for delivery; however, only a third (2187/6706) exclusively breastfed during hospital stay (Table 2). Only 15% (959/6210) of infants were exclusively breastfed for 4 months and <  1% (56/6093) were exclusively breastfed for 6 months (Table 2). Amongst the infants who were exclusively breastfed at birth and followed up to four and 6 months, the frequency of exclusive breastfeeding for four and 6 months was 49% (959/1949) and 3% (56/1902), respectively.

**Table 2:** Breastfeeding practices in the urban and semi-rural areas in Vietnam

Breastfeeding practice	Total (*n* = 6706)		Urban (*n* = 4248)		Semi-rural (*n* = 2458)		*p* value
	*n*	Frequency (%)	*n*	Frequency (%)	*n*	Frequency (%)	
Any breastfeeding during hospital stay	6706	6106 (91)	4248	3663 (86)	2458	2443 (99)	< 0.001
Exclusive breastfeeding during hospital stay	6706	2187 (33)	4248	364 (9)	2458	1823 (74)	< 0.001
Exclusive breastfeeding for 4 months	6210^a^	959 (15)	4,048^a^	70 (2)	2,162^a^	889 (41)	< 0.001
Exclusive breastfeeding for 6 months	6093^b^	56 (< 1)	3,983^b^	5 (< 1)	2,110^b^	51 (2)	< 0.001

^a^Number of infants were followed for 4 months

^b^Number of infants were followed for 6 months

All *p* - values based on Fisher’s exact test

**Fig. 1:**
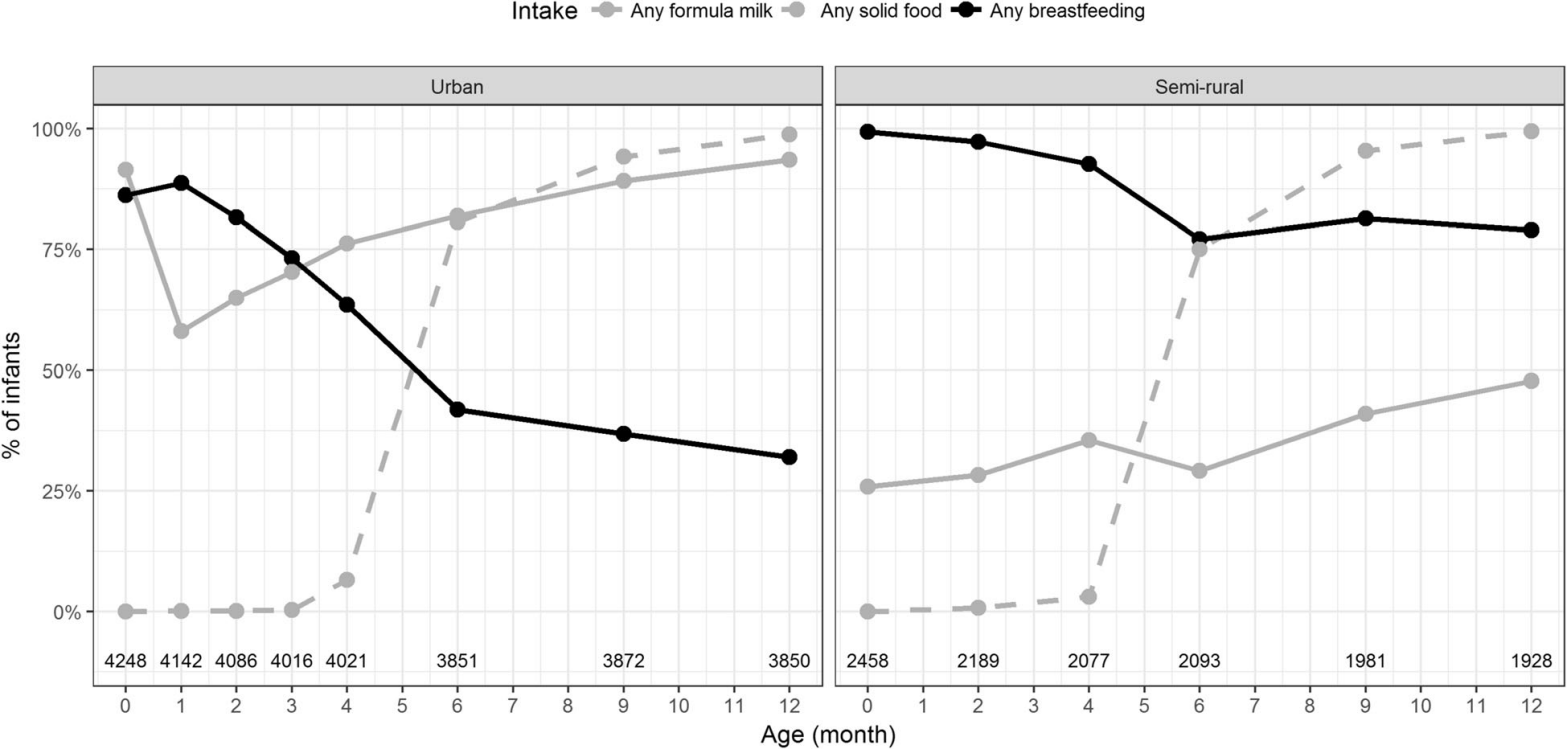
Cohort members dietary intake at routine follow-up visits during in first year of life. Plots showing the dietary intake of children in the cohort during the first year of life in the urban (left panel) and semi-rural (right panel). The dots and lines indicate the proportion for each type of intake that mothers fed their infants at each routine follow-up visit; formula milk, any solid food, and any breastfeeding (see key). Numbers at base of the are numbers of responses received at each visit

**Fig. 2:**
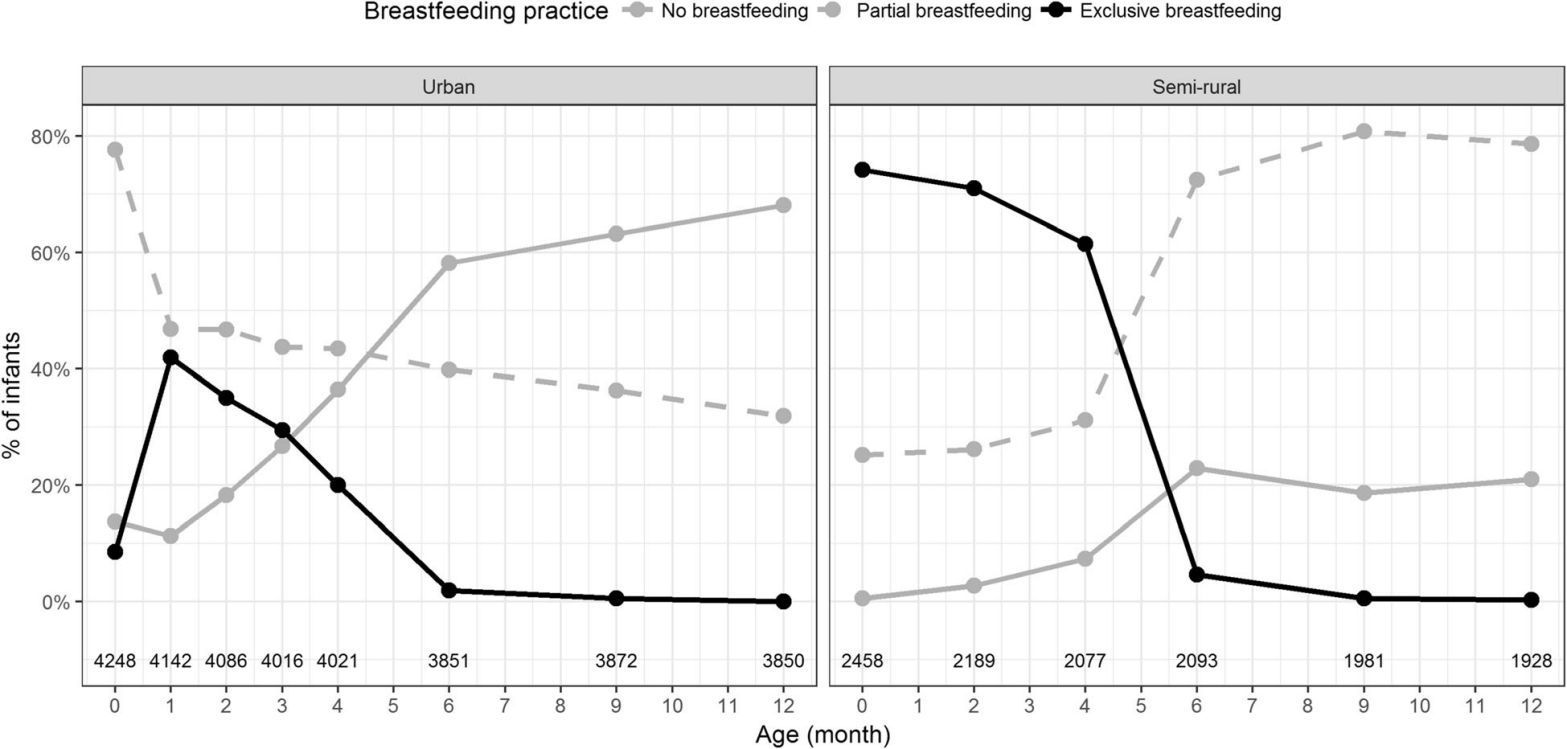
Cohort members breastfeeding practices at routine follow-up visits during the first year of life. Plots showing the breastfeeding practices in the cohort during the first year of life in the urban (left panel) and semi-rural (right panel). The dots and lines indicate the proportion for each type of self-reported breastfeeding activity at each routine follow-up visit; no breastfeeding, partial breastfeeding, and exclusive breastfeeding (see key). Numbers at base of the are numbers of responses received at each visit

In both areas, the frequency of mothers reporting any breastfeeding and exclusive breastfeeding at each of the follow-up visits decreased considerably at months four and six with the introduction of solid food (Fig. 1 and Fig. 2). In addition, most mothers reported breastfeeding their infant on at least one occasion within the first year (96%, 6222/6509 in total; 94%, 3942/4192 in urban area; 98%, 2280/2317 in semi-rural area).

The prevalence of exclusive breastfeeding during hospital stay was significantly lower in the urban population (9%; 364/4248) than the semi-rural population (74%; 1823/2458) (*p* <  0.0001, Fisher’s exact test). Similarly, all other indicators of breastfeeding practices (any breastfeeding at birth, exclusive breastfeeding for four and 6 months) were significantly lower in the urban area than the semi-rural area (Table 2). Furthermore, at the final follow-up visit (12 months after delivery), 79% (1522/1928) of mothers in the semi-rural area reported that they breastfed their children within the first year (Fig. 1). Correspondingly, 68% (2620/,3850) of mothers in the urban area reported no breastfeeding within the first year (Fig. 2). The use of infant formula was more common in the urban area, with a frequency of 58 -93%; the comparable frequency in the semi-rural area was 26 -48% (Fig. 1).

### Factors associated with exclusive breastfeeding during hospital stay

To explore factors associated with exclusive breastfeeding during hospital stay we stratified the cohort members by location (i.e. urban; *n* = 4248 and semi-rural; *n* = 2458) and conducted discrete analyses in these populations. Tables 3 and 4 show the results of univariate and multivariable analyses regarding explanatory factors associated with exclusive breastfeeding during hospital stay in the urban and semi-rural areas, respectively. In the urban population we found that mothers of higher socioeconomic status were more likely to not initiate exclusive breastfeeding during hospital stay in comparison to those in the lowest socioeconomic quintile (Odds Ratios [ORs] and 95% Confidence Interval [CIs]) for the fourth and the fifth quintiles compared to the lowest quintile: 0.38; 95% CI: 0.18, 0.88 and 0.29; 95% CI: 0.14, 0.68, respectively, *p* <  0.0001). Additionally, not initiating exclusive breastfeeding during hospital stay was associated with mothers who were currently married (OR:0.23; 95% CI 0.11, 0.50, *p* <  0.001), complications during pregnancy (OR 0.53; 95% CI 0.38, 0.74, *p* <  0.001), mothers reporting infection during pregnancy (OR 0.53; 95% CI 0.30, 0.87, *p* = 0.011), mothers delivering before week 37th of gestation (OR 0.37; 95% C: 0.15, 0.81, *p* = 0.011), mothers delivering by Caesarean section (OR 0.07; 95% CI 0.04, 0.11, *p* = 0.001), and those with neonatal complications at birth (OR 0.2; 95% CI 0.07, 0.47, *p* <  0.001).

**Table 3:** Factors associated with exclusive breastfeeding during hospital stay in the urban population

Characteristics		Yes (*n* = 364)		No (*n* = 3884)		Unadjusted			Adjusted	
	*n*	Frequency (%)	*n*	Frequency (%)	OR	(95% CI)	*p*	OR	(95% CI)	*p*
Household wealth (Ref: 1st quintile)	364		3883				< 0.001			< 0.001
2nd quintile		41 (11)		269 (7)	0.50	(0.24, 1.10)		0.71	(0.32, 1.65)	
3rd quintile		194 (53)		1463 (38)	0.43	(0.22, 0.91)		0.68	(0.33, 1.50)	
4th quintile		58 (16)		863 (22)	0.22	(0.11, 0.47)		0.38	(0.18, 0.88)	
5th quintile		60 (16)		1252 (32)	0.16	(0.08, 0.34)		0.29	(0.14, 0.68)	
Mother										
Primiparous	364	176 (48)	3883	2195 (57)	0.72	(0.58, 0.89)	0.003	0.86	(0.66, 1.12)	0.258
Age ^a^ (years)	364	28 (24, 32)	3881	28 (24, 32)	1.00	(0.98, 1.02)	0.875	1.04	(1.01, 1.06)	0.003
Ethnic (Ref: Kinh)	364		3883				0.036			0.109
Chinese		11 (3)		235 (6)	0.48	(0.25, 0.85)		0.53	(0.26, 0.98)	
Other		5 (1)		48 (1)	1.08	(0.37, 2.48)		0.74	(0.24, 1.88)	
High education	364	114 (31)	3883	1578 (41)	0.67	(0.53, 0.84)	< 0.001	1.09	(0.81, 1.46)	0.574
Currently married	364	344 (95)	3883	3827 (99)	0.25	(0.15, 0.43)	< 0.001	0.23	(0.11, 0.50)	< 0.001
In-paid employment mother	364	208 (57)	3883	2529 (65)	0.71	(0.57, 0.89)	0.003	0.81	(0.64, 1.04)	0.096
Living with others	364	344 (95)	3883	3751 (97)	0.61	(0.38, 1.01)	0.054	1.35	(0.70, 2.84)	0.386
Complication during pregnancy	364	49 (13)	3884	901 (23)	0.52	(0.37, 0.70)	< 0.001	0.53	(0.38, 0.74)	< 0.001
HIV and/or Hepatitis B infection	364	16 (4)	3884	274 (7)	0.61	(0.35, 0.98)	0.042	0.53	(0.30, 0.87)	0.011
Father										
Ethnic (Ref: Kinh)	363		3879				0.006			0.095
Chinese		27 (7)		374 (10)	0.77	(0.50, 1.14)		0.94	(0.60, 1.44)	
Other		12 (3)		45 (1)	2.85	(1.43, 5.26)		2.36	(1.08, 4.84)	
High education	363	145 (40)	3879	1786 (46)	0.78	(0.63, 0.97)	0.025	1.25	(0.94, 1.64)	0.120
In-paid employment father	363	359 (99)	3879	3851 (99)	0.65	(0.25, 2.21)	0.451	0.86	(0.29, 3.17)	0.796
Infant										
Premature at birth	364	7 (2)	3884	142 (4)	0.52	(0.22, 1.03)	0.063	0.37	(0.15, 0.81)	0.011
Male	364	187 (51)	3884	2061 (53)	0.93	(0.75, 1.16)	0.537	0.93	(0.74, 1.16)	0.510
Caesarean section	364	19 (5)	3884	1696 (44)	0.07	(0.04, 0.11)	< 0.001	0.07	(0.04, 0.11)	< 0.001
Low birthweight	364	23 (6)	3884	176 (5)	1.42	(0.89, 2.18)	0.140	2.02	(1.18, 3.33)	0.011
Neonatal complication at birth	364	5 (1)	3884	213 (5)	0.24	(0.09, 0.53)	< 0.001	0.20	(0.07, 0.47)	< 0.001

^a^described in median (interquartile)

OR: odds ratio; 95% CI: 95% confidence interval. OR, 95% CI and *p* values were estimated using univariable (unadjusted) and multivariable (adjusted) logistic regression models

High education: completed lower secondary school (> 9 years of education), Premature at birth: gestational age at birth < 37 weeks, Low birthweight: birthweight < 2500 g

**Table 4:** Factors associated with exclusive breastfeeding during hospital stay in the semi-rural population

Characteristics		Yes (*n* = 1823)		No (*n* = 635)		Unadjusted			Adjusted	
	*n*	Frequency (%)	*n*	Frequency (%)	OR	(95% CI)	*p*	OR	(95% CI)	*p*
Household wealth (Ref: 1st quintile)	1821		634				0.217			0.444
2nd quintile		803 (44)		305 (48)	0.85	(0.71, 1.02)		0.94	(0.76, 1.16)	
3rd quintile		42 (2)		14 (2)	0.97	(0.53, 1.86)		1.43	(0.71, 3.06)	
Mother										
Primiparous	1821	1085 (60)	634	407 (64)	0.82	(0.68, 0.99)	0.040	0.79	(0.62, 1.02)	0.071
Age ^a^ (years)	1822	24 (21, 29)	635	25 (22, 29)	0.98	(0.96, 1.00)	0.024	0.99	(0.96, 1.01)	0.234
Ethnic: Other (Ref: Kinh)	1820	4 (< 1)	634	1 (< 1)	1.39	(0.21, 27.30)	0.759	1.10	(0.16, 21.69)	0.929
High education	1821	394 (22)	634	167 (26)	0.77	(0.63, 0.95)	0.016	0.91	(0.69, 1.22)	0.531
Currently married	1821	1811 (99)	634	629 (99)	1.44	(0.45, 4.07)	0.517	1.19	(0.05, 43.13)	0.920
In-paid employment mother	1821	1238 (68)	634	450 (71)	0.87	(0.71, 1.06)	0.159	0.87	(0.69, 1.08)	0.203
Living with others	1819	1806 (99)	633	628 (99)	1.11	(0.35, 2.95)	0.850	0.25	(0.01, 3.56)	0.359
Complication during pregnancy	1823	212 (12)	635	97 (15)	0.73	(0.56, 0.95)	0.019	0.86	(0.65, 1.16)	0.325
HIV and/or Hepatitis B infection	1823	14 (1)	635	9 (1)	0.54	(0.23, 1.30)	0.161	0.70	(0.28, 1.93)	0.475
Father										
Ethnic: Other (Ref: Kinh)	1820	3 (< 1)	633	2 (< 1)	0.52	(0.09, 3.96)	0.488	0.57	(0.09, 4.75)	0.570
High education	1821	495 (27)	633	203 (32)	0.79	(0.65, 0.96)	0.020	0.96	(0.74, 1.26)	0.778
In-paid employment father	1821	1818 (100)	633	631 (100)	1.92	(0.25, 11.62)	0.488	2.24	(0.29, 13.85)	0.402
Infant										
Premature at birth	1823	32 (2)	635	18 (3)	0.61	(0.35, 1.12)	0.109	0.72	(0.38, 1.44)	0.346
Male	1823	925 (51)	635	330 (52)	0.95	(0.79, 1.14)	0.594	1.03	(0.85, 1.26)	0.744
Caesarean section	1823	25 (1)	635	137 (22)	0.05	(0.03, 0.08)	< 0.001	0.05	(0.03, 0.08)	< 0.001
Low birthweight	1823	75 (4)	635	35 (6)	0.74	(0.49, 1.12)	0.151	0.77	(0.50, 1.23)	0.276
Neonatal complication at birth	1823	21 (1)	635	36 (6)	0.19	(0.11, 0.33)	< 0.001	0.25	(0.14, 0.46)	< 0.001

^a^described in median (interquartile)

OR: odds ratio; 95% CI: 95% confidence interval. OR, 95% CI and *p* values were estimated using univariable (unadjusted) and multivariable (adjusted) logistic regression models

High education: completed lower secondary school (> 9 years of education), Premature at birth: gestational age at birth < 37 weeks, Low birthweight: birthweight < 2500 g

Conversely, we found that older mothers were more likely to exclusively breastfeed their infants during hospital stay (OR of exclusive breastfeeding during hospital stay for each year of age increase: 1.04; 95% CI 1.01, 1.06, *p* = 0.003). Additionally, mothers giving birth to infants with a birth weight < 2500 g were more likely to initiate exclusive breastfeeding during hospital stay (OR 2.02; 95% CI 1.18, 3.33, *p* <  0.001). In the semi-rural area, we found that delivering by Caesarean section (OR 0.05; 95% CI 0.03, 0.08, *p* <  0.001) and the infant having neonatal complications at birth (OR 0.25; 95% CI 0.14, 0.46, *p* <  0.001) was associated with lower proportion of exclusive breastfeeding in hospital (Table 4).

## Discussion

This was a large longitudinal prospective study conducted in urban and semi-rural areas in a transitional economic LMIC. We used data from this cohort to assess breastfeeding practices during the first year of life. Our results show a low prevalence of exclusive breastfeeding in hospitals immediately after birth, identifying differences in breastfeeding practices between regions with different socioeconomic structures [11, 18, 19]. Similar to previous cross-sectional breastfeeding studies conducted in Vietnam, this study confirmed that Vietnamese mothers generally consider breast milk as an important component of infant nutrition, with 94% and 98% of them breastfeeding their infant on at least one occasion in the first year after birth in urban area and semi-rural area, respectively [2]. However, the prevalence of optimal breastfeeding practices in our study was low. The rate of optimal breastfeeding practices was particularly low in the urban area, with only 9% and <  1% practicing exclusive breastfeeding during hospital stay and when the child was 6 months of age, respectively. This prevalence of exclusive breastfeeding was similar to that described in a cohort study conducted in Hong Kong in 2010 [20], but was only half the prevalence measured in Taiwan and the USA [21]. These findings suggest that promoting breastfeeding needs to be tailored to local populations.

The key finding from this study was that the factors associated with exclusively breastfeeding during hospital stay were different between urban and semi-rural areas. A greater number of factors were associated with exclusive breastfeeding during hospital stay in the urban population than the semi-rural population. In both areas, delivering by Caesarean section and having neonatal complications were strongly associated with not introducing exclusive breastfeeding during hospital stay. An explanation for these trends is the fact that current practice in obstetric hospitals in Vietnam is to separate mothers from their children after Caesarean sections and when the child has an infection, despite the benefits of early skin to skin contact [22].

We found a significantly lower prevalence of exclusive breastfeeding during hospital stay in the urban area (9%, 364/4248) in comparison to the semi-rural area (74%, 1823/2458). This figure was concordant with data from a previous cross-sectional study conducted in Hung Vuong Hospital in Ho Chi Minh City in 2014 [6], and was comparable with data originating from China regarding exclusive breastfeeding practices on hospital discharge [23]. However, a previous study from northern Vietnam, found that mothers residing in urban areas were more likely to initiate breastfeeding within 1 hour, or 1 day after birth, than women in rural areas [11]. Mothers in urban areas may correctly initiate breastfeeding within 1 hour after birth but introduce infant formula later. Our results suggest that the most common feeding practice during hospital stay in the urban area was to combine breast milk and infant formula. In the urban area, women of a higher socioeconomic status were less likely to exclusively breastfeed their infants. This difference may be linked to the capability of being able afford an alternative supplement to breast milk [9].

Participants in this study exhibited a similar pattern of breastfeeding behaviour to a population in northern Vietnam in which urban mothers in Hanoi with a higher socioeconomic status and undergoing Caesarean section were less likely to practice breastfeeding [11]. Here, the rate of delivery by Caesarean section was high (28%, 1877/6706), which was amplified in the urban setting (40; 1715/4248). This number was considerably higher than the range of 5–15% recommended by the World Health Organisation (WHO) [24]. However, this elevated figure was consistent with available data regarding the most common birthing practices in this location [6, 10, 11, 23], and in the majority of industrialized countries [25]. Potential explanations for not initiating breastfeeding in these cases are the separation of mothers and infants, a large work load for hospital staff, other priorities of healthcare professionals, and a lack of lactation consultation in large obstetric hospitals such as Hung Vuong Hospital, which has 40,000 deliveries annually [26]. Furthermore, a stressful delivery affects the initiation of breastfeeding among mothers [27], and it is perceived that antimicrobials and other drugs for the postpartum infectious have a negative impact on the benefits of breast milk [9].

Our study has limitations. As this cohort was restricted to areas with high burden of infectious diseases, this cohort may not be a population-representative sample and therefore the generalisability of our prevalence of breastfeeding practices is limited [12]. The estimated prevalence of exclusive breastfeeding for four and 6 months in the semi-rural area may be less precise than in the urban are due to differences in the number of participants recruited and in the schedules of follow up visits. Breastfeeding practices were assessed during hospital stay and follow up visits with at least one-month gap in between via interview only; therefore, these data are subject to recall bias. Investigating breastfeeding was not the primary aim of this cohort; therefore, our study criteria of exclusive breastfeeding during hospital stay, were more simplistic than those suggested by the WHO. We did not ask the time of early breastfeeding within 1 hour after birth, therefore this may induce recall bias, although time of recall was only between two and 7 days before hospital discharge. In addition, exclusive breastfeeding practices at each follow up visit were self-reported by mothers, which may lead to bias due to the different perception of the participant on the definition of exclusive breastfeeding. Nevertheless, this cohort, which used the same approach for accessing breastfeeding in this population over time [28], found that exclusive breastfeeding declines dramatically after 2 months after birth in Vietnam; identifying a specific time for the initiation of breastfeeding promotion strategies.

## Conclusions

This is one of the largest studies investigating factors associated with exclusive breastfeeding during hospital stay in Vietnam. We conclude that the practice of breastfeeding in Vietnam is a national public health issue. We have identified common risk factors associated with not initiating exclusive breastfeeding during hospital stay in urban and semi-rural areas, which included having a Caesarean section and neonatal complications. Of note, the early initiation of breastfeeding after birth in Caesarean section was incorporated into formal Vietnamese health policy, as part of the implementation of the BFHI, in 2016, [5]. Even though the BFHI was launched > 20 years ago and has been integrated into national hospital criteria since 2013, there has been no evaluation of this implementation on breastfeeding rates in Vietnam [5]. We propose future studies to investigate the most appropriate implementation strategies for improving breastfeeding in Vietnam.

## Additional file

Additional file 1: Epidemiological characteristics of participants who completed all, missed several or missed all follow-up visits. (DOCX 19 kb)

## Abbreviations

LMICs: low and middle-income countries
OxTREC: Oxford Tropical Research Ethics Committee
SES: Social Economic Status
WHO: World Health Organization
