# Supplementary material for: Factors associated with a low prevalence of exclusive breastfeeding during hospital stay in urban and semi-rural areas of southern Vietnam
Source: Int Breastfeed J. 2018 Oct 19;13:46. doi: 10.1186/s13006-018-0188-3 (PMC6194569; doi:10.1186/s13006-018-0188-3)
Supplement: Supplementary file 1 — Epidemiological characteristics of participants who completed all, missed several or missed all follow-up visits. (DOCX 19 kb) [file 13006_2018_188_MOESM1_ESM.docx]

**Additional file 1** Epidemiological characteristics of participants who completed all, missed several or missed all follow-up visits

| **Characteristics** | **Completed all FU visits**  **(*n* = 5,307)** | | **Missed several FU visits**  **(*n* = 1,202)** | | **Missed all FU visits**  **(*n* = 197)** | | ***p*-value** |
| --- | --- | --- | --- | --- | --- | --- | --- |
|  | ***n*** | **Frequency (%)** | ***n*** | **Frequency (%)** | ***n*** | **Frequency (%)** |  |
| Study site: Semi-rural | 5,307 | 1,723 (32) | 1,202 | 594 (49) | 197 | 141 (72) | < 0.001 |
| Household wealth | 5,305 |  | 1,201 |  | 196 |  | < 0.001 |
| 1^st^ quintile |  | 932 (18) |  | 311 (26) |  | 95 (48) |  |
| 2^nd^ quintile |  | 1,018 (19) |  | 342 (28) |  | 58 (30) |  |
| 3^rd^ quintile |  | 1,420 (27) |  | 268 (22) |  | 25 (13) |  |
| 4^th^ quintile |  | 801 (15) |  | 114 (9) |  | 6 (3) |  |
| 5^th^ quintile |  | 1,134 (21) |  | 166 (14) |  | 12 (6) |  |
| **Mother** |  |  |  |  |  |  |  |
| Primiparous | 5,305 | 3,102 (58) | 1,201 | 668 (56) | 196 | 93 (47) | 0.003 |
| Age ^a^ (years) | 5,304 | 27 (23, 31) | 1,201 | 26 (22, 30) | 197 | 25 (21, 30) | < 0.001^b^ |
| Ethnic | 5,305 |  | 1,200 |  | 196 |  | 0.078^c^ |
| Kinh |  | 5,048 (95) |  | 1159 (97) |  | 190 (97) |  |
| Chinese |  | 212 (4) |  | 30 (2) |  | 4 (2) |  |
| Other |  | 45 (1) |  | 11 (1) |  | 2 (1) |  |
| High education | 5,305 | 1,885 (36) | 1,201 | 343 (29) | 196 | 25 (13) | < 0.001 |
| Currently married | 5,305 | 5,236 (99) | 1,201 | 1,186 (99) | 196 | 189 (96) | 0.045^c^ |
| In-paid employment mother | 5,305 | 3,558 (67) | 1,201 | 745 (62) | 196 | 122 (62) | 0.002 |
| Living with others | 5,302 | 5,178 (98) | 1,201 | 1,164 (97) | 196 | 187 (95) | 0.056^c^ |
| Complication during pregnancy | 5,307 | 1,055 (20) | 1,202 | 181 (15) | 197 | 23 (12) | < 0.001 |
| HIV and/or Hepatitis B infection | 5,307 | 262 (5) | 1,202 | 43 (4) | 197 | 8 (4) | 0.120 |
| **Father** |  |  |  |  |  |  |  |
| Ethnic | 5,299 |  | 1,200 |  | 196 |  | 0.203^c^ |
| Kinh |  | 4,922 (93) |  | 1,120 (93) |  | 190 (97) |  |
| Chinese |  | 329 (6) |  | 67 (6) |  | 5 (3) |  |
| Other |  | 48 (1) |  | 13 (1) |  | 1 (1) |  |
| High education | 5,300 | 2,172 (41) | 1,200 | 428 (36) | 196 | 29 (15) | < 0.001 |
| In-paid employment father | 5,300 | 5,273 (99) | 1,200 | 1,192 (99) | 196 | 194 (99) | 0.334^c^ |
| **Infant** |  |  |  |  |  |  |  |
| Premature at birth | 5,307 | 150 (3) | 1,202 | 43 (4) | 197 | 6 (3) | 0.382 |
| Male | 5,307 | 2,764 (52) | 1,202 | 636 (53) | 197 | 103 (52) | 0.873 |
| Caesarean section | 5,307 | 1,603 (30) | 1,202 | 249 (21) | 197 | 25 (13) | < 0.001 |
| Low birthweight | 5,307 | 232 (4) | 1,202 | 65 (5) | 197 | 12 (6) | 0.182 |
| Neonatal complication at birth | 5,307 | 210 (4) | 1,202 | 53 (4) | 197 | 12 (6) | 0.279 |

^a^ described in median (interquartiles); All *p*-values based on Chi-squared test, except for: ^b^ based on Kruskal-Wallis test, ^c^ based on Fisher’s exact test

High education: completed lower secondary school (> 9 years of education), Premature at birth: gestational age at birth < 37 weeks, Low birthweight: birthweight < 2500g, FU = follow-up
